# Antibiotic prescribing for acute lower respiratory tract infections (LRTI) – guideline adherence in the German primary care setting: An analysis of routine data

**DOI:** 10.1371/journal.pone.0174584

**Published:** 2017-03-28

**Authors:** Eva Maria Kraus, Steffen Pelzl, Joachim Szecsenyi, Gunter Laux

**Affiliations:** 1 Darmstadt General Hospital, Department of Pharmacy, Darmstadt, Germany; 2 University Hospital Heidelberg, Department of General Practice and Health Services Research, Heidelberg, Germany; 3 SLK-Kliniken Heilbronn, Department of Pharmacy, Heilbronn, Germany; Kliniken der Stadt Köln gGmbH, GERMANY

## Abstract

**Objectives:**

Antibiotic overprescribing in primary care has major impacts on the development of antibiotic resistance. The objective of this study is to provide insight in antibiotics prescriptions for patients suffering from cough, acute bronchitis or community acquired pneumonia in primary care.

**Methods:**

Data from 2009 to 2013 of electronic health records of 12,880 patients in Germany were obtained from a research database. The prescription of antibiotics for acute lower respiratory tract infections was compared to the national S3 guideline *cough* from the German Society of General Practitioners and Family Medicine.

**Results:**

Antibiotics were prescribed in 41% of consultations. General practitioners’ decision of whether or not to prescribe an antibiotic was congruent with the guideline in 52% of consultations and the antibiotic choice congruence was 51% of antibiotic prescriptions. Hence, a congruent prescribing decision and a prescription of recommendation was found in only 25% of antibiotic prescriptions. Split by diagnosis we found that around three quarters of antibiotics prescribed for cough (73%) and acute bronchitis (78%) were not congruent to the guidelines. In contrast to that around one quarter of antibiotics prescribed for community acquired pneumonia (28%) were not congruent to the guidelines.

**Conclusions:**

Our results show that there is a big gap between guideline recommendation and actual prescribing, in the decision to prescribe and the choice of antibiotic agent. This gap could be closed by periodic quality circles on antibiotic prescribing for GPs.

## Introduction

Antibiotic resistance is a major public-health problem, in particular since resistance of microorganisms increases with the consumption of antibiotics [1]. The majority of antibiotics are prescribed in primary care, mainly for the treatment of acute respiratory tract infections [2, 3]. However, because of their limited effectiveness in only a limited number of infections, primary care guidelines recommend a restrictive use of antibiotics in respiratory infections [4].

A prospective, observational primary care study, conducted in 13 European countries, showed that clinicians could have justified an antibiotic prescription for 71.2% patients according to the guideline from the European Respiratory Society and the European Society of Clinical Microbiology and Infectious Diseases (ERS/ESCMID) with a huge variation between 30.8% in Spain and 97.2% in Hungary [5]. On the other hand, Irish investigators found that the majority (78.05%) of antibiotic prescriptions were not in accordance to national guidelines [6].

In Germany, real life information about the prescription of antibiotics in primary care is not available. In the current study we retrospectively investigated the prescription rate of various kinds of antibiotics in the primary care setting with regard to patients suffering from cough, acute bronchitis, or community acquired pneumonia.

Due to previous research, we hypothesise that physicians’ use of antibiotics will deviate from the S3 guideline *cough* from the German Society of General Practice and Family Medicine (DEGAM) for one of the following two variables: choice of prescription and choice of antibiotic.

## Methods

### Study population

The German scientific network CONTENT (CONTinuous morbidity registration Epidemiologic NeTwork), which is supported by the German State Ministry for Research (BMBF) is the basis for the present study. From January 2009 to December 2013 CONTENT cooperated with 37 resident general practitioners (GP), who participated voluntarily in this project and their practices are clustered around Heidelberg with a radius of about 70 miles. From all participating practices electronic health records of all treated patients were collected as usual care data. At the time of prescribing the GPs were not informed about the scope of our research and therefore information on diagnosis and choice of drug were recorded because of routine data. For further information see Laux et al. [7].

### Procedure

Data of patients who had one recorded acute lower respiratory tract infection (LRTI) were included in a separate database of 12,880 patients with 17,979 consultations associated with acute LRTI. Clinical diagnoses are coded using the International Statistical Classification of Diseases and Related Health Problems (ICD-10) scheme. LRTI which are included are cough (R05), acute bronchitis (J40 and J20.8) and pneumonia (J18.9) [8]. All together 7416 antibiotic items were prescribed.

According to Wood et al [5] we distinguished between ‘‘congruent prescribing”, ‘‘congruent nonprescribing”, ‘‘noncongruent prescribing”, ‘‘noncongruent nonprescribing” and whether the choice of antibiotic was “recommended” or “not recommended” [5].

Prescribed antibiotics were grouped by Anatomical Therapeutic Chemical (ATC) Classification Index [9] and the reasons for consultation were grouped by diagnosis. Both were compared to the approved S3 guideline *cough* from the German Society of General Practice and Family Medicine (DEGAM) published in 2008. We took the guideline from 2008 as a basis of our analysis because it was the current version for the observed time period. The next version was published in 2014. This guideline contains instructions for the use of antibiotics under common conditions in primary care and groups patients by risk factors. Risk factors for patients suffering from cough and acute bronchitis are: severe cardiac (ICD-10 I05-I15, I20-I28, I30-I52, I60-I79 and Q20-Q28), respiratory (ICD-10 J41-J42, J44-J47, J60-J70, J80-J86, J90-J99 and Q30-Q34) and renal (ICD-10 N00-N19, N25-N29 and Q60-Q63) diseases, cirrhosis of the liver (ICD-10 K70.3, K71.7 and K74), diabetes mellitus (ICD-10 E10-E14), congenital or acquired immunodeficiency (ICD-10 B20-B24, C00-C97 and D80-D90), and elderly patients with chronic underlying diseases (ICD-10-codes contained above). In the presence of at least one of these risk factors, antibiotic treatment is recommended for patients suffering from cough and acute bronchitis. Consultations of patients with risk factors where a prescription of antibiotic items was given were specified as “congruent prescribing”; consultations without antibiotic prescriptions were specified as “noncongruent non-prescibing”, respectively.

Patients who are younger than 65 years should receive macrolides (e.g. clarithromycin, azithromycin, roxythromycin) or tetracyclines (e.g. doxycycline). Patients who are 65 years and older should receive aminopenicillins or oral cephalosporins. If the GP chose the antibiotic-item as suggested by the guideline, the antibiotic item was grouped as “recommended choice” and as “not recommended choice” if the GP chose a different antibiotic item.

Patients without any risk factor should not receive any antibiotic item. Consequently, the group was specified as “congruent non-prescribing” if there was no antibiotic prescription. If there was an antibiotic item prescribed despite the recommendation, the consultation was grouped as “noncongruent prescribing” and the antibiotic choice was rated as for patients with risk factors. If the GP chose the antibiotic-item as suggested by the guideline, the antibiotic item was grouped as “recommended choice” and as “not recommended choice” if the GP chose a different antibiotic item.

All patients suffering from community acquired pneumonia should receive an antibiotic item according to the guideline. “Congruent prescribing” was the group of consultations with prescription of antibiotic items, and consultations without antibiotic prescriptions were grouped as “noncongruent non-prescibing”. The choice of antibiotic item depends on the existence of the following risk factors: age of 65 years and older, chronic underlying diseases (the same as mentioned for patients suffering from cough and acute bronchitis) and alcohol use disorder (ICD-10 F10-F19). Patients suffering from community acquired pneumonia without risk factors should receive an aminopenicillin (e.g. amoxicilline), a newer macrolide (e.g. azithromycin, clarithromycin, roxythromycin) or doxycycline. Patients with risk factors should receive broad spectrum penicillins + β-Lactamase inhibitor, cephalosporins or fluoroquinolones. If the GP chose the antibiotic-item as suggested by the guideline, the antibiotic item was grouped as “recommended choice” and as “not recommended choice” if the GP chose a different antibiotic item [10].

### Analysis

Relevant data were exported from Oracle MySQL Community Server (Version 5.5, 64 bit) and analysed using Microsoft Office Access® (2013). The Pearson’s chi-squared test was performed to compare categorical variables.

Moreover we assessed the decisions (right or wrong) regarding the existing guideline recommendations for the three factors

1 Consultation
2a Prescription of antibiotic
2b Choice of antibiotic

dependent on patient’s age, gender, diagnosis group (cough, acute bronchitis and pneumonia) and GP practice. We used multilevel logistic regression in order to assess these relationships. The procedure PROC GENMOD [11] of SAS (Version 9.4, x64) was applied for regression analyses.

### Ethical approval

The study’s protocol of the CONTENT project was approved by the ethics committee of the University of Heidelberg (approval number 442/2005).

## Results

Data were collected from 37 GPs located in the Heidelberg area who recorded information on 17,979 consultations. The mean (±SD) number of consultations recorded per GP was 485.92 (± 507.58). The majority of patients were females (10,692; 59.47%) and were insured by statutory health insurance (15,415; 85.74%). Some patients (936; 5.21%) changed health insurance during the duration of the study and thus cannot be grouped as statutory or private. An antibiotic was prescribed in 40% of consultations recorded (7301; 40.61%). 5% of the antibiotics (394; 5.31%) were prescribed for children between the ages of 0 and 14 years (Table 1).

**Table 1:** Demographic characteristics of the study population.

		Cough	Acute Bronchitis	Pneumonia	Total
**Age (years), Mean ± SD**		43.65 ± 26.52	45.05 ± 22.15	60.91 ± 22.91	45.70 ± 23.32
**Sex**	Male	1233 (39.19%)	5588 (40.48%)	466 (45.37%)*	7287 (40.53%)
	Female	1914 (60.82%)	8217 (59.52%)	561 (54.63%)*	10,692 (59.47%)
**Health insurance**	Statutory	2738 (87.00%)	11,828 (85.68%)	849 (82.67%)***	15,415 (85.74%)
	Private	280 (8.90%)	1212 (8.78%)	136 (13.24%)***	1628 (9.06%)
**Risk factor**	None	1661 (52.78%)	7293 (52.83%)***	285 (27.75%)***	9239 (51.39%)
	≥ 1	1486 (47.22%)	6512 (47.17%)***	742 (72.25%)***	8740 (48.61%)
**Antibiotic prescription**	Yes	131 (4.16%)***	6689 (48.45%)***	481 (46.84%)***	7301 (40.61%)
	No	3016 (95.84%)***	7116 (51.55%)***	546 (53.16%)***	10,678 (59.39%)

Data are presented as n (%) of consultations, unless otherwise stated. Chi squared test

* = P < 0.01 and

*** = P < 0.0001.

For our three analyses we could observe that there was a large variation between GP’s influence on the two dependent factors choice of prescription and choice of antibiotic, even though there are unambiguous prescribing guidelines for the considered cases.

### 1) Consultation

Looking at antibiotic prescriptions regarding different acute LRTI diagnoses, in only 4.16% (131) consultations antibiotics were prescribed for the diagnosis of cough. In nearly half of the consultations antibiotics were prescribed for acute bronchitis (6689; 48.45%) and pneumonia (481; 46.84%).

#### Guideline adherence

Regarding all 17,979 analysed consultations, at least one antibiotic was prescribed in 7301 (40.61%) consultations. Our exploratory analysis suggests that clinicians could have justified an antibiotic prescription in 9025 (50.20%) out of 17,979 consultations by a literal reading of the DEGAM guideline. In 9367 (52.10%) consultations, the decision of whether or not to prescribe was congruent with the DEGAM guideline (Table 2).

**Table 2:** Contingency table of DEGAM guideline-recommended antibiotic to be considered versus observed antibiotic prescribed for patients with acute lower respiratory tract infection.

		Antibiotic to be considered		Total
		No	Yes	
**Antibiotic prescribed**	No	5510 (30.65%)	5168 (28.74%)	10,678 (59.39%)
	Yes	3444 (19.16%)	3857 (21.45%)	7301 (40.61%)
**Total**		8954 (49.80%)	9025 (50.20%)	17,979 (100.00%)

Data are presented as n (%) of consultations. DEGAM = German Society of General Practitioners and Family Medicine

**Fig 1:**
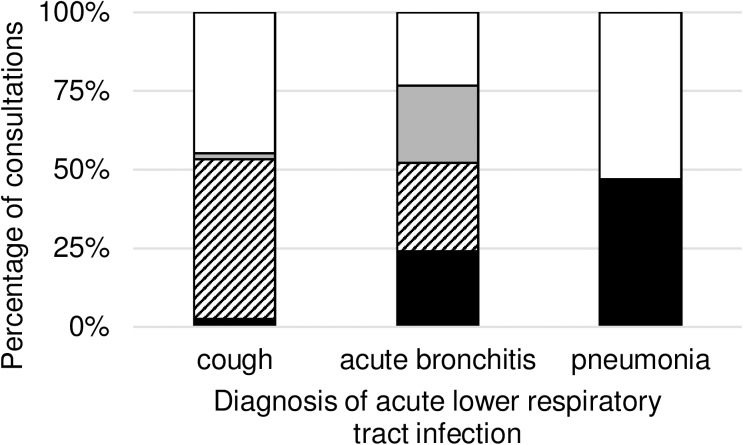
Proportion of consultations with antibiotic prescription congruence to DEGAM guideline for patients with acute lower respiratory tract infection. DEGAM = German Society of General Practitioners and Family Medicine. Consultations [%] with “congruent prescribing” of antibiotic items are shown in black, “congruent non-prescribing” are shown striped, “noncongruent prescribing” are shown in grey and “noncongruent non-prescribing” are shown white. Consultations are separated by diagnosis of acute lower respiratory tract infection.

Regarding all consultations we observed 3857 (21.45%) congruent prescribings, 5510 (30.65%) congruent non-prescribings, 3444 (19.16%) noncongruent prescribings and 5168 (28.74%) noncongruent non-prescribings. Table 3 provides information on the number and percentages of each type of prescribing split by diagnosis, and the proportion is graphed in Fig 1.

**Table 3:** Proportions of antibiotic choice congruence to DEGAM guideline for acute lower respiratory tract infection by diagnosis.

	Congruent prescribing	Congruent non-prescribing	Noncongruent prescribing	Noncongruent non-prescribing	Total
**cough**	76 (2.41%)	1606 (51.03%)	55 (1.75%)	1410 (44.80%)	3147
**acute bronchitis**	3300 (23.90%)	3904 (28.28%)	3389 (24.55%)	3212 (23.27%)	13,805
**pneumonia**	481 (46.84%)	0 (0.00%)	0 (0.00%)	546 (53.16%)	1027
**Total**	3857 (21.45%)	5510 (30.65%)	3444 (19.16%)	5168 (28.74%)	17,979

Data are presented as n (%) of consultations

The multilevel analysis on prescriptions showed that neither patient gender nor the diagnosis group had a significant influence on the right decision. Patient age was associated negatively with the right decision, but the odds ratio was close to 1 (OR = 0.989; p<0.0001; 95%-CI [0.987, 0.990]).

### 2a) Prescription of antibiotic

A total number of 7416 antibiotic items were prescribed in 17,797 recorded consultations with the diagnosis of an acute LRTI. The majority of antibiotics were prescribed for the diagnosis of acute bronchitis (6771; 91.30%), 6.89% (511) were prescribed for community acquired pneumonia and 1.81% (134) for acute cough.

#### Guideline adherence

**Fig 2:**
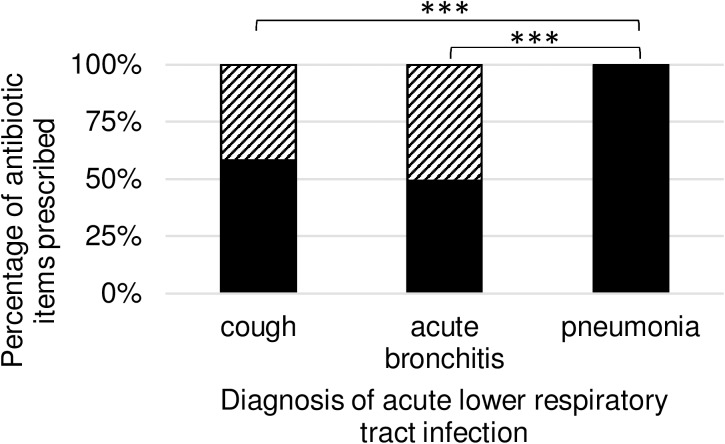
Proportions of antibiotic items with antibiotic prescription congruence to DEGAM guideline for patients with acute lower respiratory tract infection. DEGAM = German Society of General Practitioners and Family Medicine. Antibiotic items prescribed [%] with “congruent prescribing” are shown in black, and “noncongruent prescribing” are shown striped. Antibiotic items are separated by diagnosis of acute lower respiratory tract infection. Chi squared test *** = P < 0.0001

Regarding all prescribed antibiotic items we observed 3931 (53.01%) “congruent prescribing” and 3485 (46.99%) “noncongruent prescribing”. Table 4 provides information on the number and percentages of each type of prescribing split by diagnosis, and the proportion is graphed in Fig 2.

**Table 4:** Proportions of antibiotic choice congruence to DEGAM guideline for acute lower respiratory tract infection by diagnosis.

	Congruent prescribing	Noncongruent prescribing	Total
**cough**	78 (58.21%)	56 (41.79%)	134
**acute bronchitis**	3342 (49.36%)	3429 (50.64%)	6771
**pneumonia**	511 (100.0%)	0 (0.00%)	511
**Total**	3931 (53.01%)	3485 (46.99%)	7416

Data are presented as n (%) of antibiotic items prescribed. DEGAM = German Society of General Practitioners and Family Medicine

The multilevel analysis on antibiotic prescriptions showed that the decision was right for every case of pneumonia. Interestingly, the gender “male” was associated with a higher rate for right decisions (OR = 1.178; p = 0.0025; 95%-CI [1.059, 1.309]). Moreover, we observed that patient age was associated with a higher rate of right decisions (OR = 1.043; 95%-CI [1.040, 1.046]; p<0.0001). In comparison to cough, acute bronchitis was negatively associated with a right decision (OR = 0.667; p = 0.0411; 95%-CI [0.453, 0.983]).

### 2b) Antibiotic choice

Clarithromycin (1854, 25.00%), amoxicillin (1069, 14.41%), cefuroxime (800, 10.79%) and azithromycin (783, 10.56%) were the most commonly prescribed antibiotics associated with acute LRTI.

#### Guideline adherence

**Fig 3:**
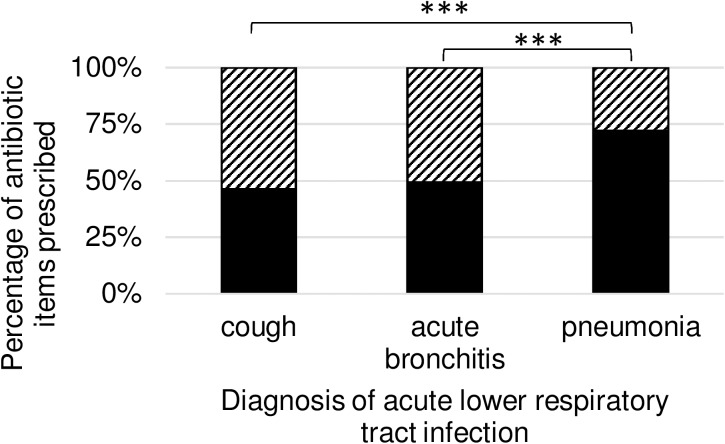
Proportions of antibiotic items with antibiotic choice congruence to DEGAM guideline for patients with acute lower respiratory tract infection. DEGAM = German Society of General Practitioners and Family Medicine. Antibiotic items prescribed [%] with “recommended choice” are shown in black, and “not recommended choice” are shown striped. Antibiotic items are separated by diagnosis of acute lower respiratory tract infection. Chi squared test *** = P < 0.0001

Regarding all prescribed antibiotic items we observed 3750 (50.57%) “recommended choice”, and 3666 (49.43%) “not recommended choice”. Table 5 provides information on the number and percentages of each type of prescribing split by diagnosis, and the proportion is graphed in Fig 3.

**Table 5:** Proportions of antibiotic choice congruence to DEGAM guideline for acute lower respiratory tract infection by diagnosis.

	recommended choice	not recommended choice	Total
**cough**	62 (46.27%)	72 (53.73%)	134
**acute bronchitis**	3320 (49.03%)	1879 (50.97%)	6771
**pneumonia**	368 (72.02%)	143 (27.98%)	511
**Total**	3750 (50.57%)	3666 (49.43%)	7416

Data are presented as n (%) of antibiotic items prescribed. DEGAM = German Society of General Practitioners and Family Medicine

The multilevel analysis on prescriptions of adequate antibiotic agents showed that patient gender and age had no significant influence on the right decision. In comparison to pneumonia, cough was negatively associated with a right decision (OR = 0.291; p<0.0001; 95%-CI [0.177, 0.478]) and acute bronchitis was even stronger negatively associated with a right decision (OR = 0.271; p<0.0001; 95%-CI [0.217, 0.338]).

### 2) Antibiotic items–prescribing decision and choice of antibiotic

1867 (47.49%) out of 3931 prescribed antibiotic items were consistent with the DEGAM guideline, and the antibiotic choice was also congruent with the DEGAM guideline (Table 6). In the group of consultations where the DEGAM guideline considers no antibiotic use (“noncongruent prescribing”), this proportion was 1883 (54.03%) out of 3485 antibiotic prescriptions.

**Table 6:** Contingency table of antibiotic choice and antibiotic prescribing decision congruence to DEGAM guideline for patients with acute lower respiratory tract infection.

		Antibiotic choice		Total
		Yes	No	
**Prescribing decision**	Yes	1867 (25.18%)	2064 (27.83%)	3931 (53.01%)
	No	1883 (25.39%)	1602 (21.60%)	3485 (46.99%)
**Total**		3750 (50.57%)	3666 (49.43%)	7416 (100.00%)

Data are presented as n (%) of antibiotic items prescribed. DEGAM = German Society of General Practitioners and Family Medicine

**Fig 4:**
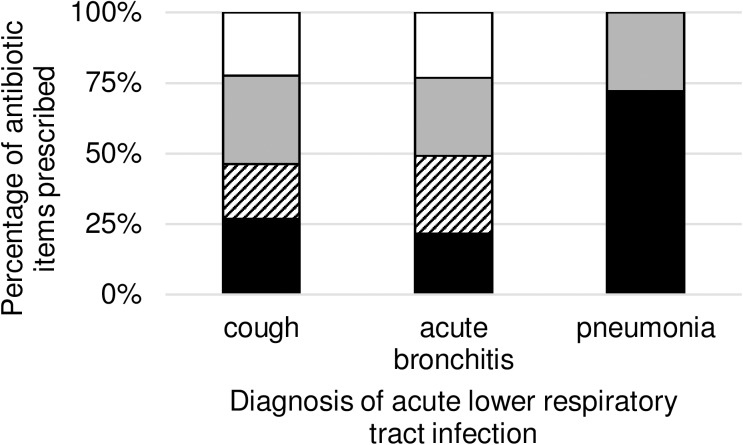
Proportions of antibiotic items with antibiotic prescription and choice congruence to DEGAM guideline for patients with acute lower respiratory tract infection. DEGAM = German Society of General Practitioners and Family Medicine. Antibiotic items prescribed [%] with “congruent prescribing, recommended choice” are shown in black, “noncongruent prescribing, recommended choice” are shown striped, “congruent prescribing, not recommended choice” are shown in grey and “noncongruent prescribing, not recommended choice” are shown in white. Antibiotic items are separated by diagnosis of acute lower respiratory tract infection.

Regarding all prescribed antibiotic items we observed 1867 (25.18%) “congruent prescribing, recommended choice”, 1883 (25.39%) “noncongruent prescribing, recommended choice”, 2064 (27.83%) “congruent prescribing, not recommended choice” and 1602 (21.60%) “noncongruent prescribing, not recommended choice”. Table 7 provides information on the number and percentages of each type of prescribing split by diagnosis, and the proportion is graphed in Fig 4.

**Table 7:** Proportions of antibiotic choice congruence to DEGAM guideline for patients with acute lower respiratory tract infection by diagnosis.

	Congruent prescribing, recommended choice	Noncongruent prescribing, recommended choice	Congruent prescribing, not recommended choice	Noncongruent prescribing, not recommended choice	Total
**cough**	36 (26.87%)	26 (19.40%)	42 (31.34%)	30 (22.39%)	134
**acute bronchitis**	1463 (21.61%)	1857 (27.43%)	1879 (27.75%)	1572 (23.22%)	6771
**pneumonia**	368 (72.02%)	0 (0.00%)	143 (27.98%)	0 (0.00%)	511
**Total**	1867 (25.18%)	1883 (25.39%)	2064 (27.83%)	1602 (21.60%)	7416

Data are presented as n (%) of antibiotic items prescribed. DEGAM = German Society of General Practitioners and Family Medicine

**Fig 5:**
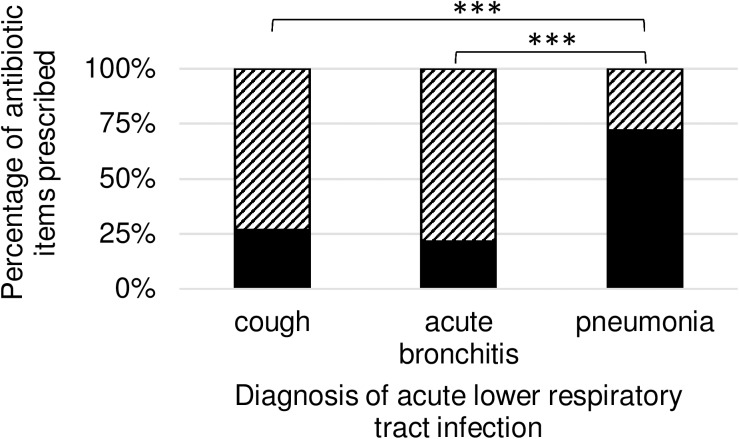
Guideline-adherence of prescriptions for antibiotic items. Antibiotic items [%] which were prescribed according to the guidelines are shown in black and non-adherence to the guidelines is shown striped. Chi-squared test *** = P < 0.0001.

The majority of antibiotic prescriptions were not in accordance to the guidelines (5549, 74.82%). Either the decision for prescribing an antibiotic item was wrong or the choice of antibiotic was not congruent to the guidelines. Nearly all of the deviations were related to the diagnosis acute bronchitis (5308, 95.66%). In “patients with cough” we found a guideline adherence of 26.87% and in “patients with acute bronchitis” the adherence was 21.61%. Interestingly, however, in “patients with pneumonia” we found nearly the reciprocal value with a guideline adherence of 72.02% (Fig 5).

## Discussion

### Summary

In the present study antibiotics were prescribed in 40.61% of consultations with the diagnosis of an acute LRTI and 74.82% of those antibiotic items were not prescribed in adherence to the S3 guideline *cough* from the German Society of General Practice and Family Medicine (DEGAM) published in 2008. Either the decision for prescribing an antibiotic item was wrong or the choice of antibiotic was not congruent to the guidelines. Detailed analysis of the three different diagnoses showed that nearly ¾ of antibiotics prescribed for cough (73.13%) and acute bronchitis (78.39%)–versus only ¼ of antibiotics prescribed for community acquired pneumonia (27.98%)–were not congruent to the guideline. For diagnosis cough and acute bronchitis these results confirm our hypothesis that physicians’ use of antibiotics will be non-congruent with S3 guideline *cough* for one of the two variables: choice of prescription or choice of antibiotic. For diagnosis pneumonia these results do not support our hypothesis.

### Strengths and limitations

The main strengths of this analysis are the big number of cases and the fact that we observed real life data and had no study conditions. Overall we analysed the data of 17,979 consultations in 37 different general practices. The data of antibiotic prescriptions were generated in the practice computers by the process of prescribing itself. At the time of prescribing the GPs were not informed about the scope of our research in order to limit any attention bias. We were able to link the information on antibiotics to the diagnosis, which helps identify inappropriately treated infections.

One limitation of this analysis is the small number of participating GPs of only 37. We want to show the prescribing practice exemplarily for one region. Routine data like this are barely not available in Germany like it is the case in other countries. As these doctors are deliberately taking part in the CONTENT project, a potential selection bias must be acknowledged. However, there is evidence that research active GPs are comparable to the wider primary care community [12]. The second potential bias might be related to GPs’ incomplete or incorrect registration of diagnostic codes and that no information on patients’ disease severity or inappropriateness of first-choice antibiotics was documented. Thus some information on patients’ factors became lost between ICD-10- and ATC-Codes and the fact that antibiotics can be clinically indicated on the basis of illness severity or patients’ anamnesis were not available for analysis. The theoretical grounds versus clinical circumstances sometimes result in deviating compliance pictures with good clinical reasons. However, LRTIs are often acute, short-term diseases for which patients contact the GP only once (as was the case in 86.00% of our consultations), therefore this bias can be negligible.

### Comparison with existing literature

For external validation the age distribution of the complete CONTENT-database in 2011 was compared to the German age distribution in 2011. The age groups between 15 and 64 years represent the German population very well. Children 14 years old and younger are underrepresented at the GP because they usually visit the paediatrician. Patients who are 65 and older are overrepresented because of their higher-than-average consultations of their GP [13]. The average age of the total population of Germany was 44.9 years in 2011 [14]. In this study the average age was 45.7 years. In 2011 41.4% of German patients in general practices were male [15]. The difference to our study population is only 1%. In the total population of Germany in 2011 85.1% were insured by statutory health insurance and 11.0% privately [16]. In our study population 85.7% were insured by statutory health insurance and 9.1% by private health insurance. All these demographic characteristics show that the study population represents the total German population very well, even if the sample is very small.

In the present study antibiotics were prescribed in 40.61% consultations with the diagnosis of an acute LRTI. Related to this finding a 13 European country observational study showed that 52.7% patients consulting primary care with acute cough/LRTI received a prescription of antibiotics [5]. On the other hand, a cross sectional observational study with clinicians from a primary care research network in Germany showed that 34.50% of adults with a new or worsening cough or a clinical presentation that suggested a LRTI received an antibiotic treatment [17]. The difference to our analysis is that the mentioned two studies were performed under study-conditions. Our study adds real life data to these results. The decision of whether or not to prescribe an antibiotic was congruent to the guideline in 52.10% of consultations. The 13 European country observational study displayed a similar result: in 50.3% of consultations GPs’ decisions were right [5]. Our multilevel analysis showed a negative association of patient age and the right decision. The odds ratio was so close to 1 (OR = 0.989) that the result is indeed significant but not relevant. A Dutch analysis found that potential over-prescribing of antibiotics for RTIs occurs in the age group 31–65 years, not in children and the elderly [18].

Regarding all prescribed antibiotic items the decision of prescription was right for 53.01% of antibiotics. There is a wide difference to 80.3% which were found for Germany in the 13 European country observational study [5]. Furthermore, the European study is a result of different countries and we observed only one country. Our multilevel analysis showed that the gender “male” was associated with a higher rate for right decision (OR = 1.178). A Swedish analysis also found a gap between perceived and expressed knowledge of sex and gender differences in drug treatment [19]. We observed that higher patient age was associated with a higher rate of right decisions (OR = 1.043) which is contrary to the result of consultations but in accordance to the Dutch analysis [18]. The odds ratio was also close to 1 (OR = 0.989) indicating that the result is indeed significant but not relevant. Relevant is the fact that in comparison to cough, acute bronchitis was negatively associated with the right decision (OR = 0.667). Patients with diagnosis of acute bronchitis are more likely to receive an antibiotic in opposition to guideline recommendations than patients with diagnosis of cough. A different result was found in the Netherlands: Overprescription in patients with acute cough was 70% and in patients with acute bronchitis 69% [20]. But results from different countries are not comparable as there is a huge variability [17].

In the present study we identified that 74.82% of all antibiotic items prescribed for acute LRTIs were not prescribed in adherence to the S3 guideline *cough* from the German Society of General Practice and Family Medicine (DEGAM) published in 2008. Irish Investigators published in 2012 that the majority of antibiotic prescriptions were not strictly in accordance to guidelines (78.05%) in consultations associated with the respiratory system [6]. The 13 European country observational study showed the opposite outcome: clinicians could have justified an antibiotic prescription for 71.2% patients by a literal reading of the ERS/ESCMID guidelines. However, after performance of a sensitivity analysis the overall percentage reduced to 29.7%, resulting the not-adherence to be 70.3% [5]. Detailed analysis of the three different diagnoses showed that nearly ¾ of antibiotics prescribed for cough (73.13%) and acute bronchitis (78.39%)–versus only ¼ of antibiotics prescribed for community acquired pneumonia (27.98%)–were not congruent to the guideline. Having nearly the reciprocal value to prescriptions for cough and acute bronchitis, the value of antibiotics prescribed for community acquired pneumonia is very surprising. The reason might be the increased attention clinicians might give to patients suffering from the life-threatening community acquired pneumonia as it is a very profound decision to treat a patient ambulatory [10]. Our multilevel analysis on prescriptions of adequate antibiotic agents gets to the same result as the univariate analysis regarding the diagnosis. Patient age and gender had no influence.

The reasons behind the failure to adhere to the guidelines are discussed in some previous literature. In one study it was acknowledged that the guideline recommendation had even been unknown to some of the GPs and therapeutic decisions were mainly driven by what was perceived as prevailing practice [21]. Livorsi et. al. identified three barriers to guideline-concordant care: physicians’ lack of awareness of specific guideline recommendations; tension between adhering to guidelines and the desire to individualise patient care; and scepticism of certain guideline recommendations [22].

## Conclusions

This paper presents an analysis of routine data on antibiotic prescribing in primary care in Germany regarding acute LRTIs. Inappropriate prescribing is a problem and, given the impact that antibiotic prescribing has on antimicrobial resistance, it is important that the prescribing behaviour is explored. Our data show, that in contrast with the mostly guideline congruent pneumonia therapies, antibiotics are vastly overprescribed for cough and acute bronchitis. There is a need to focus on reducing antibiotic use for cough and acute bronchitis and increasing the use of first-choice antibiotics.

We would like to add that we do not regard all non-adherence cases as wrong treatment decisions, as guidelines are not laws and GPs are not computers. However, we recommend that the effects of guidelines are actively monitored to demonstrate the benefits and safety of national implementation of prescribing advice. In the German federal state Baden-Württemberg, where the study was performed, there are periodic quality circles on different topics of pharmacotherapy for GPs. Our findings strictly suggest to consistently address inadequate antibiotic prescribing within these quality circles.

## Supporting information

S1 Data Study data. (ZIP)

## References

[1] Goossens H, Ferech M, Vander Stichele R, Elseviers M, Group EP. Outpatient antibiotic use in Europe and association with resistance: a cross-national database study. Lancet. 2005;365(9459):579–87. 10.1016/S0140-6736(05)17907-0 15708101

[2] van den Broek d'Obrenan J, Verheij TJ, Numans ME, van der Velden AW. Antibiotic use in Dutch primary care: relation between diagnosis, consultation and treatment. J Antimicrob Chemother. 2014;69(6):1701–7. 10.1093/jac/dku005 24508898

[3] Petersen I, Hayward AC, Subgroup SS. Antibacterial prescribing in primary care. J Antimicrob Chemother. 2007;60 Suppl 1:i43–7. 17656380 10.1093/jac/dkm156

[4] Smith SM, Fahey T, Smucny J, Becker LA. Antibiotics for acute bronchitis. Cochrane Database Syst Rev. 2014;3:CD000245. 10.1002/14651858.CD000245.pub3 24585130

[5] Wood J, Butler CC, Hood K, Kelly MJ, Verheij T, Little P, et al Antibiotic prescribing for adults with acute cough/lower respiratory tract infection: congruence with guidelines. Eur Respir J. 2011;38(1):112–8. 10.1183/09031936.00145810 21233267

[6] Murphy M, Bradley CP, Byrne S. Antibiotic prescribing in primary care, adherence to guidelines and unnecessary prescribing—an Irish perspective. BMC Fam Pract. 2012;13:43 10.1186/1471-2296-13-43 22640399 PMC3430589

[7] Laux G, Koerner T, Rosemann T, Beyer M, Gilbert K, Szecsenyi J. The CONTENT project: a problem-oriented, episode-based electronic patient record in primary care. Inform Prim Care. 2005;13(4):249–55. 16510021 10.14236/jhi.v13i4.604

[8] (KKG) AIdKfFdKiG. Internationale statistische Klassifikation der Krankeheiten und verwandter Gesundheitsprobleme. Deutsches Institut für Medizinische Dokumentation und Information (DIMDI); 2014.

[9] (WIdO) G-AiWIdA. Anatomisch-therapeutisch-chemische-Klassifikation mit Tagesdosen—Amtliche Fassung des ATC-Index mit DDD-Angaben für Deutschland im Jahre 2015. In: Gesundheit Bf, editor.: Deutsches Institut für Medizinische Dokumentation und Information (DIMDI); 2015.

[10] Braun V. Husten. (DEGAM) DGfAuF, editor. Düsseldorf: Deutsche Gesellschaft für Allgemeinmedizin und Familienmedizin (DEGAM); 2008. 1–80 p.

[11] SAS. 9.4 ed: PROC GENMOD Statement.

[12] McManus RJ, Ryan R, Jones M, Wilson S, Hobbs FR. How representative of primary care are research active practices? Cross-sectional survey. Fam Pract. 2008;25(1):56–62. 10.1093/fampra/cmm065 18048650

[13] Statistisches Bundesamt. Wiesbaden2016 [Available from: https://www.destatis.de/DE/ZahlenFakten/GesellschaftStaat/Bevoelkerung/Bevoelkerungsstand/Tabellen/AltersgruppenFamilienstandZensus.html.

[14] SeBaWorld SB. Welt auf einen Blick Berlin2002-2013 [cited 2017 07.02.2017]. Available from: www.welt-auf-einen-blick.de/bevoelkerung/durchschnittsalter.php.

[15] ISEG. Barmer-GEK-Arztreport 2013. St. Augustin; 2013.

[16] Gottfried M. vdek-Basisdaten des Gesundheitswesens 2012/2013. Berlin; 2012.

[17] Butler CC, Hood K, Verheij T, Little P, Melbye H, Nuttall J, et al Variation in antibiotic prescribing and its impact on recovery in patients with acute cough in primary care: prospective study in 13 countries. BMJ. 2009;338:b2242 10.1136/bmj.b2242 19549995 PMC3272656

[18] Akkerman AE, van der Wouden JC, Kuyvenhoven MM, Dieleman JP, Verheij TJ. Antibiotic prescribing for respiratory tract infections in Dutch primary care in relation to patient age and clinical entities. J Antimicrob Chemother. 2004;54(6):1116–21. 10.1093/jac/dkh480 15546973

[19] Loikas D, Karlsson L, von Euler M, Hallgren K, Schenck-Gustafsson K, Bastholm Rahmner P. Does patient's sex influence treatment in primary care? Experiences and expressed knowledge among physicians—a qualitative study. BMC Fam Pract. 2015;16:137 10.1186/s12875-015-0351-5 26462960 PMC4603906

[20] Dekker AR, Verheij TJ, van der Velden AW. Inappropriate antibiotic prescription for respiratory tract indications: most prominent in adult patients. Fam Pract. 2015;32(4):401–7. 10.1093/fampra/cmv019 25911505

[21] Kuehlein T, Goetz K, Laux G, Gutscher A, Szecsenyi J, Joos S. Antibiotics in urinary-tract infections. Sustained change in prescribing habits by practice test and self-reflection: a mixed methods before-after study. BMJ Qual Saf. 2011;20(6):522–6. 10.1136/bmjqs.2010.047357 21262789

[22] Livorsi D, Comer AR, Matthias MS, Perencevich EN, Bair MJ. Barriers to guideline-concordant antibiotic use among inpatient physicians: A case vignette qualitative study. J Hosp Med. 2016;11(3):174–80. 10.1002/jhm.2495 26443327 PMC4779411

